# Novel RNAi delivery systems in the control of medical and veterinary pests

**DOI:** 10.1016/j.cois.2019.02.001

**Published:** 2019-08

**Authors:** Miranda MA Whitten

**Affiliations:** Institute of Life Science, Swansea University Medical School, Singleton Park, Swansea, SA2 8PP, UK

## Abstract

RNA interference (RNAi) is a transformative technology with great potential to control, study or even protect insects and acarines through the knockdown of target gene expression. RNAi offers unprecedented levels of control, but fundamental to its successful deployment is the need to deliver ‘trigger’ RNA in an appropriate fashion giving due consideration to potential barriers of RNAi efficiency, safety, and the intended purpose of the knockdown. This short review focusses on recent innovations in RNAi delivery that are designed for, or could be adapted for use with, insect and acarine pests of medical or veterinary importance.

**Current Opinion in Insect Science** 2019, **34**:1–6

This review comes from a themed issue on **Vectors and medical and veterinary entomology**

Edited by **Claudio R Lazzari** and **Anna Cohuet**

For a complete overview see the Issue and the Editorial

Available online 12th February 2019

**https://doi.org/10.1016/j.cois.2019.02.001**

2214-5745/© 2019 Elsevier Inc. All rights reserved.

## Introduction

The underlying principles of arthropod RNAi and the common hurdles limiting RNAi efficiency encountered by insects have been reviewed comprehensively elsewhere [e.g. 1, 2, 3, 4, 5, 6]. Key to maximum RNAi efficiency is the capacity to avoid or circumvent the dual perils of nuclease activity (in the hemolymph and especially the gut) and extremes of gut pH, both of which can degrade or destabilize introduced ‘trigger’ interfering RNAs (double-stranded RNA – dsRNA; short hairpin RNA – shRNA; short interfering RNA – siRNA) [1,7,8]. Ideally, trigger RNA should arrive intact at its target cell, whereupon it is readily taken up (e.g. via clathrin-mediated endocytosis or via scavenger receptors) and escaping the endosomal system [9] to be efficiently diced into siRNAs before entering the RNAi pathway. Even more preferably, the RNAi effect acts systemically and with subsequent propagation (amplification) since from a pest control perspective, systemic RNAi is more likely to impart a lethal phenotype.

**Figure 1:**
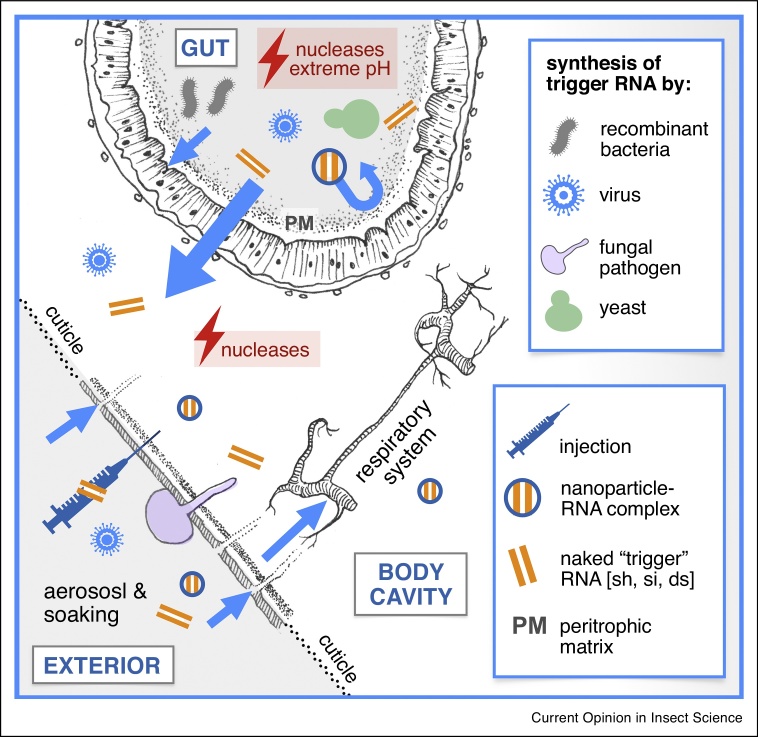
Key delivery systems for inhibitory ‘trigger’ RNA in insects. Common anatomical sites of RNA entry are indicated together with delivery vehicles and modes of entry. The introduced trigger RNA must avoid degradation or deactivation by nucleases and extreme pH in the gut and hemolymph. The small pores of the gut peritrophic matrix may further represent a physical barrier to larger delivery vehicle particles such as nanoparticles.

Two factors define RNAi delivery strategies: (i) the anatomical site of entry for trigger RNA, and (ii) in what form the RNA is to be administered. Common sites of entry are the gut, the respiratory system, the cuticle – either through traumatic penetration or via epicuticular uptake, and *in situ* synthesis by live microbes – usually in the gut but potentially in any body compartment (Figure 1). As will be discussed below, many RNAi delivery vehicles are equally capable of entering the insect body at multiple entry sites, but not necessarily equally effectively. With the exception of mosquitoes, pests of medical and veterinary significance are underrepresented in RNAi research, probably because of the challenges posed in rearing and maintaining them for study in a laboratory environment. However, most of the delivery systems described below have the potential to be adapted to medical and veterinary pests.

## Ingestion

Recent RNAi experiments with the sarcoptic mite *Sarcoptes scabiei* [10^•^] and the house dust mite *Dermatophagoides pteronyssinus* [11] have utilized total immersion in dsRNA over several hours, with significant gene knockdown. Some of the RNA is ingested, as indicated by the appearance of fluorescently labelled RNA in the gut. The authors envisage eventually developing therapeutic dsRNA-based topical treatments against mite infestations. The cat flea *Ctenocephalides felis*, a pest of considerable medical and veterinary significance, is the first siphonapteran in which RNAi has been demonstrated recently [12^•^]. Microinjection, ingestion and soaking were compared as dsRNA delivery methods, and although all three methods were impressively effective, ingestion via a bloodmeal elicited the strongest knockdown of a sigma-class GST antioxidant gene. Although it is not clear whether each method administered a comparable dose, the injections delivered a fairly minimal 69 ng per insect. Gut nucleases do not appear to be problematic in this insect; it is also interesting that the authors remarked on the surprisingly good stability of dsRNA mixed into bovine citrated blood, in contrast to the observations of Basnet and Kamble [13].

## Cuticular penetration

Injection of dsRNA remains an essential tool in many RNAi proof-of-principle studies that can inform future pest control strategies. Some recent examples of successful RNAi deployment by injection highlight targets for the suppression of fecundity. These include knockdown of *vitellogenin* in adult female bedbug *Cimex lectularius* [14] in which the phenotype persisted for several weeks, *RpATG6* in Chagas disease vector *Rhodnius prolixus* adult females (to disrupt yolk production) [15], and *ribosomal protein S6* in the adult female housefly *Musca domestica* [16]. Of note are the variable quantities of dsRNA used in these studies, ranging from 20 ng (bedbug; roughly equivalent to 2.7 ng per mg body mass) to 5 μg (housefly; approximately 238 ng per mg body mass).

Systemic RNAi has already been demonstrated in all developmental stages of several tick species, and immersion of whole ticks in aqueous solutions of dsRNA targeting the essential gene *Hlfer1* are effective (e.g. [17]). Zhang *et al.* [18] refined this approach by combining fluorescently labelled dsRNA with a liposome transfection agent to determine the best uptake mechanism in ticks. The resulting pattern of fluorescence was consistent with direct uptake through the pores and canaliculi of the epicuticle, and the mouth. They observed that the target *P0* gene was most effectively knocked down by prolonged soaking (17 hours) with liposome–RNA complexes in all lifecycle stages, and superior to aqueous solutions. Interestingly the most important factor determining RNAi efficiency was the duration of soaking, being more critical than concentration or liposome type.

A few studies have attempted topical uptake by administering dsRNA mixed with acetone – in which it is stable – directly to the insect cuticle. This was first trialled with adult *Aedes aegypti* mosquitoes in 2008 [19] using dsRNA directed against the *Diap1* homologue *AeIAP1*. A re-evaluation of the paper in 2016 [20] indicated that it was not possible to conclude that topical application was successful. It is unclear whether the application itself was ineffective or whether the problem rested with the gene target; however, injection of dsRNA targeting IAPs failed to produce convincing phenotypes in three mosquito species [20] except when incubated with Aag2 cells. Topical application of dsRNA targetting *actin* (in either acetone or water) was also unsuccessful in bed bug nymphs [13] despite ds*actin* eliciting a measurable knockdown by injection. Galay *et al.* [17] observed that *Haemaphysalis longicornis* ticks, unlike *Varroa* mites, are not susceptible to dsRNA soaking when it is dissolved in 0.9% NaCl, while they are very sensitive when the dsRNA is in aqueous solution and possibly taken up osmotically; temperature was also an important parameter. This interesting observation cautions that we need to understand the biochemistry and composition of the integument in order to design the best medium for soaking.

## Cross-kingdom RNAi: delivery by microbes

### Bacteria

Trigger RNA has been expressed in recombinant *Escherichia coli* for many years, and is often subsequently fed to target insects to elicit RNAi; however, in most insects *E. coli* can also stimulate an immune response. The use of symbiotic or commensal bacteria to synthesize dsRNAs *in insecta* is a natural progression from *E. coli*-based dsRNA expression and the paratransgenesis principle [21,22]. Prolonged or even indefinite knockdown can be achieved with symbionts because the manipulated RNaseIII-deficient bacteria are re-introduced to their natural insect host where they establish a continuous turnover of dsRNA [23^•^]. This system has been developed in *Rhodococcus rhodnii* (the symbiont *R. prolixus*) in which stable dsRNA expression is afforded by integrating the dsRNA expression cassette into the bacterial chromosome [23^•^]. Short-term RNAi was also demonstrated in *R. prolixus* using dsRNA hairpin-expressing *R. rhodnii* with a presumably intact *rnaseIII* [24^•^]. In common with some viruses (see below) symbiont delivery systems theoretically offer two-tier specificity (that of the dsRNA sequence, and host-symbiont co-evolution), and the opportunity to exploit natural transmission through an insect population by horizontal or vertical transmission [25]. A caveat is that endosymbionts with a long history of host co-evolution are not culturable in the lab.

### Viruses and virus-like particles

*In situ* synthesis of trigger RNAs can also be achieved through viruses and this strategy was reviewed extensively by Kolliopoulou *et al.* [26]. Recent developments (in *Drosophila*) have built on an approach first used successfully by Gu *et al.* [27], who created a recombinant mosquito-specific densovirus (AeDNV) expressing shRNA against the essential *V—ATPase* gene. Taning *et al*. [28^•^] have now created a recombinant Flock House virus (FHV) capable of producing siRNAs. Taning *et al*. approach with the multi-host FHV was to create a tool for functional studies in a wider variety of RNAi-recalcitrant insects, which could in future include pests of medical significance. The team transfected *Drosophila* S2 cells with two engineered plasmids containing a capsid protein precursor gene and the RNA-dependent polymerase gene and a *Drosophila melanogaster* target sequence for dsRNA production during viral replication. These were expressed in the cytoplasm, and the two combined to make infectious virions. For pest control, an oral infection method with purified virions would be most appropriate but a very high dose is needed for the infection of insects such as drosophiliids [29] and mosquitoes, and the potential host range of the virus and its mutation rate would need to be risk assessed.

There are inherent risks with the use of any live microbes repurposed to synthesize trigger RNAs, including recombination events. Persistent virus infections may also present specific problems if they do not turn over sufficient quantities of interfering RNAs. Alternatively, viruses can subvert a normally robust RNAi system by saturating the binding capacity of the RNAi pathway and/or by inhibiting key molecules in that pathway [30]. However, there are interesting exceptions such as Israeli acute paralysis virus, which actually appears to enhance the RNAi response in its bumblebee host [31,32].

Virus-like particles (VLPs) could be a more practical solution that avoids many of the problems with infective virions. These are spontaneously self-assembled structural components of viruses that encapsidate small RNA molecules. Although there appear to be no recent examples of VLP development in insect RNAi, a good overview of the technology can be found in Ref. [26].

### Yeast

*Anopheles gambiae* larvicides consisting of yeast expressing specific shRNAs have been developed recently [33^••^]. This is a very promising approach for insects with aquatic larval stages. The authors identified three putative larval essential genes *Sac 1*, *otk*, *lrc* and then used yeast expression vectors to express shRNAs in baker’s yeast *Saccharomyces cerevisiae,* with lethal RNAi phenotypes after ingestion. Even dead yeast cells proved to be effective larvicides, so the group formulated dried, inactivated yeast RNAi tablets, which makes them suitable for administering cheaply to remote areas of mosquito endemicity. This study is interesting too because, unusually, shRNAs were synthesized in preference to long dsRNAs to ensure a level of specificity (since all shRNA sequences are known) that cannot be guaranteed with dsRNA.

### Entomopathogenic fungi

A recombinant dsRNA-expressing strain of the entomopathogenic fungus *Isaria fumosorosea* has been developed to infect the whitefly, *Bemisia tabaci* via cuticlar penetration and then knock down an immunity gene *TLR7* [34,35^••^]. The synergistic approach of using a fungal pathogen to enhance its own virulence via RNAi chimes with the wider concept that RNAi is probably most effectively deployed in concert with other pest control strategies. Intriguingly, the authors speculated that it may be the initial immune attack itself that damages the invading blastospores, thus liberating the dsRNA [34]. While *I. fumosorosea* lacks host-specificity, other species and strains of entomopathogenic fungi are available that exhibit greater specificity (e.g. [36]) and these could in future be exploited to deliver RNAi to disease vectors such as mosquitoes.

## Nanoparticles

Nanoparticles (NPs) represent an exciting and dynamic area of innovation in RNAi delivery technology. In entomology, chitosan (a polysaccharide from arthropod exoskeletons), silica, perfluocarbon, guanidine-containing polymers and carbon quantum dot NPs have all been complexed with trigger RNA. These have been administered variously in insect cell culture [37], by feeding [37, 38, 39, 40, 41,42^••^,43^••^], topical application [18] and even by aerosol [44,45^•^]. When chosen appropriately, NPs not only stabilize dsRNA, shRNA and siRNA and shield the RNA from nucleases and extremes of pH, but they also optimize cellular uptake. The practical steps in NP synthesis are often surprisingly straightforward, and NPs are created with a positive charge to enable binding to negatively charged RNA. Most NPs also offer the advantage of being biodegradable and/or biocompatible. It is, however, important to note that not all NPs are universally appropriate; silica-based NPs perform poorly at extremes of pH and degrade in the strongly alkaline condition the mosquito larval midgut, while carbon quantum dots are highly effective [37]. Chitosan–dsRNA complexes in *Aedes* larvae also perform well [37,41]. Particle size should also be considered if ingested NPs are intended to traverse the peritrophic matrix [46].

Perfluocarbon-bound siRNA NPs have been administered by aerosol to aphids [45^•^]. The aim was to stabilize the RNA trigger and deliver it to internal organs via the tracheoles thereby bypassing the gut and, to some extent, the hemolymph. Aerosolization of naked RNA improved RNAi efficiency, and even more so with siRNA–NP complexes. Thairu *et al*. method [45^•^] was based on a ground-breaking study by Li-Byarlay *et al*. [44] which exposed adult worker honeybees for 5 min. to nebulized aerosols of PFC-NP-siRNA. It is tempting to speculate that such an approach could be used to kill *Varroa* mites and it could lend itself to treating social and colonial insects that congregate in confined spaces.

Cationic polymethacrylate derivative polymer NPs containing guanidine have recently been successfully trialled for oral delivery of dsRNA in two notoriously RNAi-recalcitrant species of *Spodoptera* (Lepidoptera) [42^••^,43^••^]. Polymers with a high guanidine content form stable complexes with dsRNA at high pH and thus protect it from the hostile gut environment. They also exhibit enhanced cellular uptake by mimicking arginine-rich cell penetrating peptides. These NPs would be applicable to the anterior midgut of *A. gambiae* and *A. aegypti* larvae at pH 11 [47]. Transfection agent ‘lipoplexes’ have also been used to protect dsRNA in the gut of the cockroach *Blatella germanica* to allow local (not systemic) knockdown of an essential midgut-expressed *tubulin* gene [40]; however, lipoplexes may not be able to permeate the peritrophic matrix.

## Conclusions

Despite the meteoric rise in popularity of CRISPR-cas9 genome editing tools, RNAi remains a popular and important reverse genetics strategy for entomological research and for potential applications in pest control. RNAi is envisaged not as a standalone pest control tool but rather as part of a synergistic approach. Critical to the success and safety of insect-targetting RNAi is the appropriate choice of delivery method. At present the most exciting innovations are the use of nanoparticles, and the expression of trigger RNAs by a range of microbes. These offer some practical solutions for deployment in the field for pest control, therapeutics, and even possibly for targeting pathogens in vectors rather than the vectors themselves. Challenges lie ahead to minimize and risk-assess off-target silencing and the potential for RNAi resistance to emerge.

## Conflict of interest statement

Nothing declared.

## References and recommended reading

Papers of particular interest, published within the period of review, have been highlighted as:

• of special interest
•• of outstanding interest
